# Single Cell Biology: Exploring Somatic Cell Behaviors, Competition and Selection in Chronic Disease

**DOI:** 10.3389/fphar.2022.867431

**Published:** 2022-05-17

**Authors:** Wandi Zhu, Rahul C. Deo, Calum A. MacRae

**Affiliations:** Cardiovascular Medicine Division and Department of Medicine, Brigham and Women’s Hospital, Harvard Medical School, Boston, MA, United States

**Keywords:** clonal hematopoeisis, cell competition, chronic inflammation, single cell physiology, therapeutics

## Abstract

The full range of cell functions is under-determined in most human diseases. The evidence that somatic cell competition and clonal imbalance play a role in non-neoplastic chronic disease reveal a need for a dedicated effort to explore single cell function if we are to understand the mechanisms by which cell population behaviors influence disease. It will be vital to document not only the prevalent pathologic behaviors but also those beneficial functions eliminated or suppressed by competition. An improved mechanistic understanding of the role of somatic cell biology will help to stratify chronic disease, define more precisely at an individual level the role of environmental factors and establish principles for prevention and potential intervention throughout the life course and across the trajectory from wellness to disease.

## Introduction

Infectious disease is perhaps the most obvious and acute form of cell “competition” in human experience, with innate and adaptive immune responses collaborating to eliminate exogenous cells which themselves are participants in the competition. Related biology also drives the clearance of aberrant native cells, such as damaged or neoplastic cells. These processes require activation and consumption of the immune system, generally described as the inflammatory response (Baldridge et al., 2010; King and Goodell 2011). A substantial component of the variation in inflammatory responses between individuals is thought to reflect inherited differences in immune reactivity or the overt effects of chronic comorbidities (Initiative 2021). The discrete outcomes from identical insults among superficially similar individuals are a manifestation of our limited ability to detect meaningful intrinsic differences in many biological processes with current tools. Looking at orthogonal outputs of these same immune or inflammatory pathways may be useful as these cellular functions also play central roles in defining hierarchies in distinctive cell non-autonomous processes including synaptogenesis, metabolism, and aging (Lee et al., 2014; Sekar et al., 2016).

Accumulating evidence shows that individual variation in inflammatory responses may also be influenced by lifelong competition between different somatic cell populations, some of which arise as they acquire cell behaviors with differential advantages in various tissues. Such competitions among somatic cell populations represent potentially modifiable components of many chronic diseases (Thorpe et al., 2020). In this model, the emerging dominance of particular cellular populations may result from active selection at many levels including specific functional attributes which create advantage, differential rates of proliferation in dividing cells or simple survival in terminally differentiated cells (Nagata and Igaki 2018; Lawlor et al., 2020).

The preponderance of genetic selection in evolution antedates multicellularity, so that cell competition is a useful framework for understanding emerging mechanisms of somatic selection and for understanding the net outputs of competing cell behaviors (Maruyama and Fujita 2021; Swiatczak 2021). In this short perspective, we will summarize existing evidence of contributions from distinctive somatic cell populations to chronic disease, highlight those few mechanisms that have been established, outline the constraints which limit detection of somatic selection, and illustrate the potential of scalable single cell biology to explore the dynamics of cell competition throughout the course of many common diseases. We will focus on inflammatory biology, but the mechanisms and investigative approaches we discuss are likely pertinent in many other areas.

## Mechanisms of Cell Competition

The specification of cell behaviors during development, the general patterning of the body plan, tissue architecture, and even long-term features of cell states, are dependent on stereotypic cellular events prespecified by transcriptional programs and interactive cues from neighboring or remote cells (Bruce and Zernicka-Goetz 2010; Zhu and Zernicka-Goetz 2020; Maruyama and Fujita 2021). These cues operate in pathways that share overlapping mechanisms with single cell organisms, and have been conceptually aggregated under the framework of cell competition. The outcomes of cellular competition are dependent on the baseline fitness or dynamic responses of participating cells and the prevailing conditions under which competition takes place. Together, the combination of innate cell behaviors, acquired functional properties, and the extrinsic selection forces determine the discrete outcomes of cell competition.

The spectrum of pathways implicated in cell competition includes those with obvious mechanisms, as well as others where the distinctive cellular advantages conferred may be more obscure (Baker 2020). Clearly, the efficiency of cell division varies among cells and those capable of more rapid cell division or improved use of metabolic resources fare better throughout development (Lawlor et al., 2020). The ribosome is a complex molecular machine that is responsible for protein synthesis. The biogenesis of ribosome is one of the most energy demanding cellular processes and it is strongly associated with cell survival and proliferative capacity. In cell or organismal screens, gene variation affecting ribosomal biogenesis has consistently been found to be among the most important differences between successful and vanquished cells. Not surprisingly, strong selection bias is also observed with mutants in genes encoding core drivers of cell division itself, such as the tumor suppressors *Myc* and T*p53* (Madan et al., 2018; Nagata and Igaki 2018). Why tumor suppressors more generally do not exhibit effects on developmental cell competition is not obvious from extant experimental data. It is possible there may be strong evolutionary advantages to carefully balanced competition in most tissues, particularly in epithelia. Indeed, intercellular coupling in epithelia is related to cellular competition in a complex manner. Coupling can average differences across multiple cells, for example in the concentrations of intracellular metabolites or second messengers (Kiselyov et al., 2003). This effect can synchronize cell signaling across entire cellular compartments, but also can be a direct sensor of gradients of competitiveness particularly with metabolic substrates or toxins. These aspects may be reflected in the apicobasal cell polarity genes identified in cell competition screens (Nagata and Igaki 2018). Selection based on fitness or stochastic processes is also a key component of the formation of discrete cellular networks from neuronal circuits to antigen processing (Willis et al., 2006; Costa-Rodrigues et al., 2021). The ultimate fate of the ‘losers’ in such cellular population competitions is not often studied, though it may include different forms of programmed cell death, new local cell fates, or escapes to new futures. Indeed, the extrusion of less competitive cells is one means by which cell competition has been identified (Maruyama and Fujita 2021). Some extruded cells undergo apoptotic cell death, while others may initiate epithelial-mesenchymal transitions (EMTs) or revert to stem cell-like fates (Ohsawa et al., 2018). The same pathways are exploited in normal development and throughout adult life where travelling cellular offspring may leave progenitor compartments for new environments.

Cell competition signals are also central to cellular maturation and adaptation in multiple contexts from early development to aging (Alvarez-Dominguez and Melton 2022). In chronic disease, it has recently become evident that cellular competition and the balance between germline or somatic variation and the environment is playing out at many levels, potentially explaining significant components of the interindividual variation in the progression, responses to therapy and outcomes of highly prevalent disorders.

## Insights From Clonal Hematopoietic Abnormalities

Early insights into the role of cell competition in chronic disease emerged from the study of human genomics, where spontaneous somatic mutation with proliferative advantage became detectable as genotyping technologies enabled deeper characterization of variation from germline in DNA samples collected from peripheral blood. Accessible cells in the periphery are dominated by representatives from the hematopoietic compartment which includes the majority of cells that govern inflammation (King and Goodell 2011). Hematopoiesis provides a distinctive setting to investigate cell competition during adulthood, as the system generates hundreds of billions of cells across multiple lineages every day throughout life. To maintain this output, the hematopoietic system has adopted a hierarchical differentiation scheme that allows enormous amplification from a few hematopoietic stem cell (HSC) clones (Orkin and Zon 2008). As part of the inflammatory response, HSCs are exposed to cues that signal specific adaptations and the production of new immune cells (Medzhitov 2008; King and Goodell 2011), which may accelerate the acquisition of somatic mutations. The emergence of dominant HSC clones is partly due to the effects of mutations on fitness advantages for proliferation or survival in the bone marrow environment (Watson et al., 2020). However, studies also suggest HSCs possessing mutations remain small clones in the bone marrow for many years and are present in healthy individuals (Mendez-Ferrer et al., 2020). It is plausible that a shift in bone marrow microenvironment, the accumulation of additional mutations or a change in the effective selection pressures is necessary to initiate clonal expansion. For example, a recent study showed that the expansion of mutant HSCs is driven by their resistance to inflammatory signals, driven by their mature cell progeny (Avagyan et al., 2021). Besides the hematopoietic lineages, DNA isolated from peripheral blood may also contain nucleic acids from other sources including endothelial cell “progenitors,” neoplastic cells from multiple tissues, chimaeric fetal cells, subcellular exosomes and cell-free nucleic acids (Szilagyi et al., 2020; Avramovic et al., 2021). The complexity of these DNA sources also impacts our interpretation of the mechanisms of cell competition in chronic disease (Avramovic et al., 2021).

Seminal work from Ebert’s group first identified the presence of age-related clonal hematopoietic abnormalities in peripheral blood, distinct from the myelodysplastic syndrome but associated with increased risk of adverse outcomes including hematologic cancer, incident stroke, coronary heart disease and all-cause mortality (Jaiswal et al., 2014). The detected clones shared a series of underlying mutations in a small number of genes that are recurrently mutated in hematological neoplasia, including *DNMT3A*, *TET2* and *ASXL1*. Subsequent work has defined a growing number of associations between this phenomenon, known as clonal hematopoiesis of indeterminate potential (ChiP), and a wide range of other chronic disease outcomes including heart failure, atrial fibrillation, chronic obstructive lung disease, osteoporosis, transplant outcomes, and severe COVID-19 (Jaiswal et al., 2017; Dorsheimer et al., 2019; Dawoud et al., 2021; Feusier et al., 2021).

The underlying mechanisms for such associations are not yet evident and are likely to differ among clones and among chronic diseases. In at least some of these settings it appears that CHiP may be an index of prior exposures, presumably driving mutagenesis or selection (Coombs et al., 2017; Dawoud et al., 2020). There is evidence that some forms of CHiP are a reflection of a shared upstream susceptibility to a range of disorders mediated by different hematopoietic lineages (Bick et al., 2020). It is quite consistent with existing data that in many situations CHiP represents a sophisticated index of environmental genotoxicity or of inflammatory risk *per se* but is not necessarily a mechanistic driver. Only mechanistically targeted interventional studies will directly address these uncertainties.

Mouse models have demonstrated the potential importance of clonal hematopoietic behaviors in non-neoplastic phenomena including lesion formation in atherosclerosis. TET2 null progenitor clones expand in a chimeric transplant and also drive substantial increases in lesion size in the LDLR homozygous null model of atherosclerosis (Fuster et al., 2017). JAK2-mutant clones are associated with the highest levels of risk for atherosclerotic events among human CHiP cohorts. In murine models of these same JAK2 mutations (V617F), expressed selectively in macrophage lineages, increased proliferation of macrophages and prominent formation of necrotic cores in atherosclerotic lesions was observed. These changes can be suppressed by loss of function alleles in inflammasome components or by deletion of gasdermin D or AIM2 (Fidler et al., 2021). These experiments reinforce existing concepts of atherosclerosis progression and support a role for CHiP in the exacerbation of endovascular inflammation. Nevertheless, it remains difficult to fully recapitulate the likely decades-long effects of interactions between clones where only the victors ultimately manifest in the genomic analyses. Until there are comprehensive models of the lifelong competition between clones and the variable influences of other intrinsic or environmental factors on various stages of disease progression, it will prove difficult to define the relative contributions of different cellular mechanisms to the final outcomes. Once again, understanding the role of the losers may be at least as important as characterizing the winners in clonal competitions.

One tantalizing insight into the potential determinants of the final outcomes in complex and dynamic cellular competitions is the observation that the presence of CHiP was related to the observed effects of IL1β inhibition (with the monoclonal antibody canukinumab) on incident coronary events and on incident lung cancer in the rigorous CANTOS trial (Ridker et al., 2017; Svensson et al., 2022). This response to therapy may be a consequence of the particular features of the inflammatory response in these individuals, but also suggests there may be much broader roles for CHiP as an index or driver of potential outcomes in a wide range of disease-relevant and therapy-relevant cellular competitions.

As sequencing depth increases, evidence is accumulating from genotyping of peripheral blood and tissue samples from broader populations that somatic variation is a universal phenomenon. The initial identification of CHiP mainly focused on genes that have been previously implicated in causing hematologic malignancy. It has become clear that those genes may only underlie a limited portion of clonal selection (Zink et al., 2017). Examples are emerging of somatic mutations in single genes or even at single residues underlying very specific clinical syndromes. For example, somatic mutations in the X-chromosomal ubiquitinylation enzyme, UBA-1, have been shown to cause an adult onset autoinflammatory disorder in males through activation of the innate immune system as the underlying clone becomes dominant (Beck et al., 2020).

Unassayed somatic variation and underlying clonal competition must be considered as potential contributors in all genotype-phenotype associations (Avramovic et al., 2021). It will require serial genotyping and/or single cell analysis of genotypes to deconvolute fully the contributions of clonal competition to the large number of established genetic associations, and at present genotyping depth is the rate-limiting step in the detection of clonal imbalance. In addition, evidence is now emerging that dynamic non-genetic selection with sustained effects on cell behavior is contributing to the progression of chronic disease and may also be more widespread than previously imagined.

## Functional Selection

The existence of cellular competition between clones with a definable genomic basis for winning or losing, also implies a need for single cell genomic and functional analyses to understand those clonal attributes selected or lost in the competition. In addition genomics (even single cell genomics) may offer insufficient resolution to detect cell population shifts in the setting of epigenetic differences due to prior exposures or in the context of small or isogenic clones. There is now emerging definitive evidence of the existence of active cellular competition and selection based on functional attributes resulting not from genetic variation between clones, but rather from acquired ‘fitness’ under specific environmental constraints. In the absence of genetic mechanisms and consequent effects on allelic distributions, such selection may only be detected using single cell analyses. Together these observations suggest that single cell genotypic and phenotypic characterization of somatic cellular populations will be an important step in improving our understanding of the relationship between genotype and phenotype as well as the discrete and dynamic contributions of cell competition to the trajectories of common diseases.

Functional selection may operate through multiple mechanisms and is also likely to be more readily modifiable than intrinsic genotype-based clonal imbalance. All the factors that influence clonal behaviors can bias cellular, tissue or organ physiology in the absence of any changes in the genotypes, numbers, or capabilities of the underlying cell populations. For example, a period of exposure to low oxygen tension (PaO_2_) may result in an erythrocyte cohort with dysregulated expression of erythropoietin (EPO) and hypoxia inducible factors (HIFs), with potential consequences for numerous disease states through the life cycle of these specific erythrocytes. Systematically classifying, across multiple phenotypic dimensions, single cell responses to disease, drug and other “environmental” exposures will enable the development of mechanistic insights into clonal and non-clonal behaviors underpinning individual variation in disease biology.

## Evidence of Environmental Selection

There is extensive evidence that environmental factors including stress, pollution and determinants of health all influence both acute and chronic inflammation, though in most instances the molecular mechanisms are not known or are incompletely understood. With the increasing accessibility of single cell biology (Gradeci et al., 2020), in particular in immunophenotyping, there is accumulating evidence of the direct effects of even modest changes in acquired or environmental factors on integrated cellular responses.

### Salt and Autoimmunity

Work in mouse has elegantly defined the effects of modest increases in salt intake on the architecture of T-cell responses. The TH17 phenotype of helper T cells was known to be stabilized and reinforced by IL-23 signaling. Transcriptomic studies of the development and differentiation of the TH17 subset revealed a dependence on the salt inducible serum glucocorticoid kinase 1 (SGK1) (Wu et al., 2013). Genetic studies in mouse revealed a requirement for SGK1 as a mediator of the effects of high salt concentrations on TH17 production and on consequent inflammation. Increased dietary salt intake was also shown to lead to a more severe phenotype in murine autoimmunity models through the induction of a population of pathogenic and highly stable TH17 cells (Kleinewietfeld et al., 2013). These studies demonstrate regulation of T cell clonal selection process by dietary salt intake that ultimately impacts the autoimmune response and promotes tissue inflammation.

### Smoking and Clonal Hematopoiesis

Smoking triggers immunological responses and alters inflammatory markers, such as C-reactive protein (CRP), fibrinogen, and leukocyte count, due to its direct effects on macrophage, neutrophil, and dendritic cell activity, as well as activation of subsequential immune responses due to epithelial and endothelial cell damage caused by reactive oxygen species. Analyses of human whole genome and whole exome sequencing data established a strong association between smoking and the occurrence of clonal hematopoiesis. The proinflammatory environment caused by smoking promotes the outgrowth of ASXL1-mutant hematopoietic stem cell clones (Dawoud et al., 2020). Moreover, smoking is linked to hematopoietic clonal expansion in absence of mutations in the traditionally defined driver genes, such as *TET2*, *DNMT3A*, *ASXL1*, and *PPM1D* (Zink et al., 2017), suggesting that environmental factors may drive cell competition without basal predisposition to somatic mutation.

### Hyperglycemia, Thrombosis and Piezo1

Mechanical cues from the local environment or neighboring cells regulate cell behaviors and competitive interactions. Ion channels in the cell membrane, including the mechanosensory ion channel Piezo1, transduce mechanical cues into rapid electrical signals that control and amplify many downstream cellular functions. While the roles of ion channels are well-defined in excitable cell types, such as cardiomyocytes and neurons, their functions in non-excitable cells remain poorly understood. Recent studies reveal that ion channels are expressed abundantly on the cell membranes of peripheral blood cells and regulate processes including adaptive and innate immune responses, platelet activation, and red blood cell homeostasis (Cahalan et al., 2015; Feske et al., 2015; Ma et al., 2018). Besides somatic genetic alterations, ion channel expression and function may also be modulated by the environment or by chronic disease.

**FIGURE 1:**
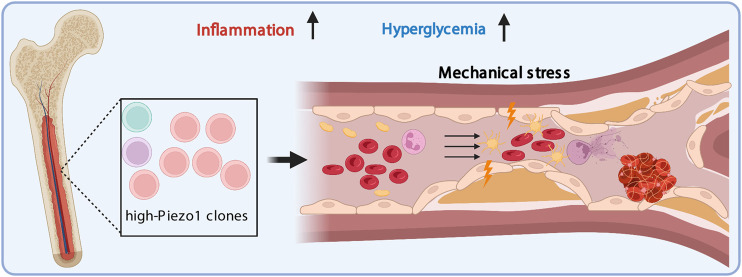
Cell competition related to PIEZO1 during hematopoiesis. PIEZO1 expression levels lead to selection at several levels. High PIEZO1 expressing cells have proliferative advantage at baseline which is accentuated by hyperglycemia at key stages of hematopoiesis. This advantage may be determined by genetics, epigenetics or even stochastic variation. Subsequent selection may include survival advantages in multiple other settings each of which may apply only to specific cell types, but the net outcome is an associated increase in the susceptibility to thrombosis in a significant proportion of diabetic patients. Detecting such variation requires single cell phenotyping at scale.

Thrombosis is the leading complication of common human disorders including diabetes, coronary heart disease, cancer, and infection. In a focused screen for single cell ion channel phenotypes associated with disease, it was possible to demonstrate a direct role for the mechanosensory ion channel Piezo1 in clot formation in subjects with type 2 diabetes (T2DM) that is triggered by disordered blood flow (Zhu et al., 2022). Piezo1 function and expression are exquisitely controlled by the glycemic environment and in T2DM Piezo1 was upregulated in multiple blood lineages. In both *ex vivo* HSC differentiation and *in vivo* zebrafish whole kidney marrow transplantation, there was evidence that exposure to an elevated glucose concentration enriched high-PIEZO1-expressing HSCs and enhanced HSC differentiation towards pro-inflammatory monocytes and dendritic cells. The net effects of this modulation of mechanosensory channel expression and function in T2DM were a distinctive prothrombotic state which could be suppressed by specific inhibition of the Piezo1 channel. Subsequent studies in human samples confirmed a Piezo1-dependent prothrombotic state in a significant proportion of patients with T2DM. Findings from this study implicate the hyperglycemic environment in HSC clonal expansion and differentiation based on underlying variation in mechanosensitivity, which substantially contributes to elevated thrombotic risk (Figure 1). This modulation may be further impacted by changes in mechanical properties of the bone marrow microenvironment due to aging and chronic diseases.

Together these examples suggest that general environmental exposures or acquired microenvironmental features of chronic diseases exert selection pressure during hematopoiesis and lead to distinctive pathophysiologic courses. The detection and modulation of such effects in chronic diseases may open new therapeutic strategies, while the exploration of such single cell biology in simple blood samples highlights the possibility for scalable translational studies to define the dynamic effects of acquired factors on cellular physiology (Gradeci et al., 2020).

## Systematic Characterization of Human Cell Population Function

**FIGURE 2:**
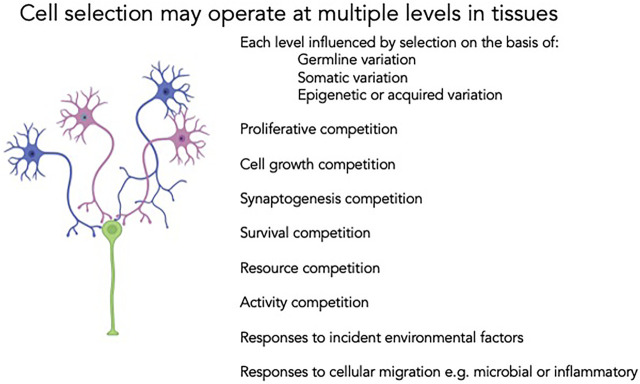
Cell competition is also feasible in terminally differentiated cell lineages. Neurons may undergo selective pressures at multiple stages of their life cycle. Proliferative advantages for individual clones may be reinforced or counteracted in subsequent competition for synaptogenesis with other cells in neuronal circuits, activity-based functional inputs or differential susceptibility to ischemia, inflammation or other external pressures. The net output of the system, and the penetrance and expressivity of germline genotype, is a reflection of each of these levels of selection. A more rigorous appraisal of the discrete steps and the operant selection pressures will enable improved diagnosis, prognostication and therapy.

Cell biology offers critical insights into the dynamic interactions between genes and environment but has long been relatively inaccessible in clinical and translational investigation. Human cell biology has been studied in only a few clinical settings; excised neoplastic tissue, liquid tumors and immunophenotyping, all typically at modest scale. Even in these contexts, the collection of single cell functional data is limited, though interest in circulating tumor cells and the falling costs of flow cytometry are slowly impacting the field. Unbiased approaches to even simple cellular data can predict clinically useful disease outcomes (Patel et al., 2015), and the association of CHiP and somatic genetic variation with discrete risk for a wide range of pathologies further highlights the need for deeper mechanistic insight. To understand fully the diversity of cellular mechanisms at play in somatic cell behavior, and their contributions to human responses to the environment, to drugs or to acquired components of chronic disease, there is a need for much broader exploration of single cell function and single cell genomics in clinical cohorts. In addition, to understand the dynamic responses even in terminally differentiated cells it will be important to assess biology under baseline and perturbed conditions (See Figure 2).

## Emerging Technologies and Future Directions

How can cell biology be implemented at a scale similar to that of population genomics? The use of simple accessible technologies in large clinical populations would enable efficient expansion of the collection of clinically annotated cell biological data. An initial focus on widely available tools including morphometry, microscopy and vital dyes could identify disease areas where deeper exploration of cell function might be of utility. Using cellular data, computational approaches, including deep learning, can facilitate the extraction of relevant functional response signatures and identify discrete and previously unobserved cell populations or behaviors. Microfluidic systems, single cell capture methodologies and a range of cellular resolution phenotypes will enable much more granular exploration of the basic functional abnormalities in disease states. Expanding the capabilities of such tools to include exosomes and other circulating cell-derived particles with their contents will open a window into non-circulating cells in health and disease. Combining these assays to discern the features associating with specific cellular states will offer novel insights into the inflammation and other chronic disease mechanisms.

Ultimately, it will be important to characterize the full range of cell states and behaviors in health and disease. Exploring cell biology and its effects on cell population dynamics will likely offer mechanistic insights which are pertinent far beyond the specific cell types that are assayed. For instance, a combination of single cell transcriptional profiling, spatial, and temporal tracking has been applied to reveal dynamic and complex immune cell populational behaviors at the site of inflammation. There are now numerous examples of peripheral cellular phenotypes revealing biology of impact in remote and inaccessible cell types such as podocytes, adipocytes or even cardiomyocytes (Genovese et al., 2010; Asimaki et al., 2016). Scalable approaches to single cell function are available and will complement the genomic tools developed over the last 2 decades. Indeed, without additional phenotyping granularity, it will be difficult to realize mechanistic insights at the level of the individual patient, a prerequisite for the full potential of precision medicine. Cellular studies will also offer direct insight at a personal level into environmental or acquired factors.

The full range of cell functions is one of the most underdetermined aspects of disease biology. The evidence that somatic clonal imbalance and cell competition play a role in non-neoplastic chronic disease reveals the need for a dedicated effort to explore single cell function to understand disease mechanisms. It will be vital to document not only the prevalent pathologic behaviors but also those beneficial functions eliminated or suppressed by competition. Phenotype expansion in this way to define the roles of somatic cell biology will help to stratify chronic disease, annotate the human genome in regular clinical encounters, and define quantitative contributions from genes and environment across the lifecourse.
